# Tomato seed bio-priming with *Pseudomonas aeruginosa* strain PAR: a study on plant growth parameters under sodium fluoride stress

**DOI:** 10.3389/fmicb.2023.1330071

**Published:** 2024-01-04

**Authors:** Anamika Singh, Anil Patani, Margi Patel, Suhas Vyas, Rakesh Kumar Verma, Abdelfattah Amari, Haitham Osman, Lokendra Rathod, Noureddine Elboughdiri, Virendra Kumar Yadav, Dipak Kumar Sahoo, Rajendra Singh Chundawat, Ashish Patel

**Affiliations:** ^1^School of Liberal Arts and Sciences, Mody University of Science and Technology, Sikar, India; ^2^Department of Biotechnology, Smt. S. S. Patel Nootan Science and Commerce College, Sankalchand Patel University, Visnagar, India; ^3^Department of Life Sciences, Hemchandracharya North Gujarat University, Patan, India; ^4^Department of Life Sciences, Bhakta Kavi Narsinh Mehta University, Junagadh, Gujarat, India; ^5^Department of Chemical Engineering, College of Engineering, King Khalid University, Abha, Saudi Arabia; ^6^ICMR-National Institute for Research in Environmental Health, Bhopal, India; ^7^Chemical Engineering Department, College of Engineering, University of Ha’il, Ha’il, Saudi Arabia; ^8^Chemical Engineering Process Department, National School of Engineers Gabes, University of Gabes, Gabes, Tunisia; ^9^Department of Veterinary Clinical Sciences, College of Veterinary Medicine, Iowa State University, Ames, IA, United States

**Keywords:** PGPR, *Pseudomonas aeruginosa* strain PAR, fluoride stress, tomato, antioxidant enzymes

## Abstract

The primary goal of this experiment is to examine the effectiveness of *Pseudomonas aeruginosa* strain PAR as a rhizobacterium that promotes plant growth in mitigating the negative effects of fluoride-induced stress in tomato (*Lycopersicon esculentum* Mill.) plants. A total of 16 rhizobacterial strains were tested for plant growth-promoting (PGP) attributes, with isolates S1, S2, and S3 exhibiting different characteristics. Furthermore, growth kinetics studies revealed that these isolates were resilient to fluoride stress (10, 20, 40, and 80 ppm), with isolate S2 exhibiting notable resilience compared to the other two strains. Phylogenetic analysis revealed isolate S2 as *P. aeruginosa* strain PAR. Physiological analyses demonstrated that *P. aeruginosa* strain PAR had a beneficial impact on plant properties under fluoride stress, comprising seed germination, root length, shoot height, relative water content, and leaf area, the strain also impacted the buildup of glycine betaine, soluble sugar, and proline, demonstrating its significance in enhancing plant stress tolerance. In *P. aeruginosa* strain PAR-treated plants, chlorophyll content increased while malondialdehyde (MDA) levels decreased, indicating enhanced photosynthetic efficiency and less oxidative stress. The strain modified antioxidant enzyme action (catalase, ascorbate, glutathione reductase, peroxidase, and superoxide dismutase), which contributed to improved stress resilience. Mineral analysis revealed a decrease in sodium and fluoride concentrations while increasing magnesium, potassium, phosphorus, and iron levels, emphasizing the strain’s significance in nutrient management. Correlation and principal component analysis revealed extensive correlations between physiological and biochemical parameters, underscoring *P. aeruginosa* strain PAR’s multifaceted impact on plant growth and stress response. This study offers valuable information on effectively utilizing PGPR, particularly *P. aeruginosa* strain PAR, in fluoride-contaminated soils for sustainable agriculture. It presents a promising biological strategy to enhance crop resilience and productivity.

## 1 Introduction

A multitude of microorganisms inhabit the rhizosphere and provide beneficial effects to plants by restraining the infiltration of harmful pathogens and aiding in the absorption of nutrients from the soil (Leach et al., 2017; Patani et al., 2023a). Based on several experiments conducted, it can be asserted that the host plant plays a crucial role in determining the composition of its rhizosphere microorganisms. The plant rhizosphere supports a diverse variety of microbes, including archaea, bacteria, nematodes, fungi, viruses, and protists (Ling et al., 2022; Qin et al., 2022; Sun et al., 2022). Plant rhizospheres usually have a wide variety of soil microorganisms. It is indeed a fact that a certain group of PGPRs have the ability to significantly aid plant growth promotion. These PGPRs are typically recognized as plant growth-promoting rhizobacteria that are supposed to hold immense potential as an eco-friendly substitute to chemical/inorganic fertilizers (Mahanty et al., 2016; Bhattacharyya et al., 2020; Li et al., 2022). The PGPR predominantly includes members from genera such as *Beijerinckia*, *Arthrobacter*, *Burkholderia*, *Ochrobactrum*, *Derxia*, *Herbaspirillum*, *Klebsiella*, *Pantoae*, *Acinetobacter*, *Enterobacter*, *Rhodococcus*, *Gluconacetobacter*, *Bacillus*, *Alcaligenes*, *Acetobacter*, *Arthrobacter*, *Azospirillum*, *Pseudomonas*, *Lactobacillus*, *Azotobacter*, *Stenotrophomonas*, *Zoogloea*, *Paenobacillus*, *Azoarcus*, and *Serratia* (Vega-Celedón et al., 2021; Li et al., 2023).

PGPR either directly or indirectly helps the general well-being of the related plants. Plant growth can be enhanced through the process facilitated by PGPR, which includes nitrogen fixation, solubilization of zinc, potassium, and phosphate in the soil, provision of iron to the host plant through siderophore synthesis, and generation of phytohormones (Mahanty et al., 2016; Patani et al., 2020; Orozco-Mosqueda, et al., 2023; Patani et al., 2023b, Patel et al., 2023a,b).

Choudhary et al. (2007) demonstrated that PGPR uses various indirect mechanisms to protect plants from stressors, including the synthesis of hydrolytic enzymes and exo-polysaccharides and promoting induced systemic resistance (ISR). It also aids in heavy metal bioremediation. PGPR effectively enhances plant cell responses to external stimuli. As a result, it induces systemic resistance and activates the plant immune response, leading to a more robust and resilient plant. This mechanism works by priming the plant to respond more effectively to potential threats, such as pests or pathogens. By optimizing the plant’s response to external stimuli, PGPR helps to improve plant health and productivity while reducing the need for harmful chemicals and pesticides. PGPR has frequently been discovered to boost the synthesis process of chemicals related to defense in the host plants (Kumar and Verma, 2018; Singh et al., 2023).

Zhang et al. (2019) discovered a connection between fluoride levels and bacterial community composition in shallow groundwater in northern China’s Qiji region. In groundwater, the volume of total organic carbon (TOC) and fluoride have a considerable effect on bacterial growth. When examining fluoride-rich groundwater systems and the associated environmental conditions, it is important to take into account the various biogeochemical processes at play, particularly those related to groundwater and the concentration of fluoride. By doing so, it is possible to more accurately assess the microbial response and the overall impact of these conditions and defensive systems can be developed by PGPR in response to fluoride toxicity. Mukherjee et al. (2017) discovered *Providencia vermicola* KX926492, a fluoride-resistant bacterium from severely contaminated rural water, and demonstrated a maximum fluoride remediation of 82%. These microbes can endure and sustain higher fluoride concentrations, offering an effective way to reduce the toxicity of fluoride. By altering the fluoride antiporter’s promoter, rhizobacteria can become resistant to fluoride stress (Liao et al., 2016).

Pelc et al. (2020) revealed that winter wheat cultivars exhibited a decrease in their ability to germinate and a suppression of their root growth. Additionally, catalase activity inhibition was observed in both the roots and embryos of these cultivars. They also identified that fluoride levels have increased and that the concentration of sodium fluoride (NaF) has a bigger impact on the activity of antioxidant enzymes than the cultivar of winter wheat; therefore, fluoride contamination diminishes plant yield, which eventually affects human food sources. These findings highlight the importance of rhizobacteria in sustainable agriculture and their potential as biofertilizers.

Fluoride contamination in agricultural soils is a major threat to crop yield, necessitating novel and long-term stress reduction strategies. There are several companies that develop chemicals for controlling pests and provide alternative sources of essential nutrients, such as macro and micronutrients. These sectors also exert a significant impact on the proliferation of hazardous compounds in the environment and food products. Fluoride is one of the pollutants that are harmful to both plants and humans (Zuo et al., 2018). Multiple harmful substances are causing a decline in the well-being of soil, plants, and animals. One such substance is fluoride, which occurs naturally in soil and is commonly found in groundwater worldwide. In India, the highly fluoride-affected states are Rajasthan, Gujarat, and Andhra Pradesh, where the fluoride concentration is higher than >1.5 ppm, and these areas are known as fluoride endemic areas (Choubisa et al., 2022). The problem of elevated fluoride levels is crucial due to toxicological and geo-environmental issues. There is an urgent need to develop robust rhizobacterial species to mitigate the harmful consequences of fluoride accumulation in plants (Aggarwal and Bhushan, 2019; Chatterjee et al., 2020). Tomato is the most commonly utilized vegetable crop among horticultural products, and it ranks first among canned vegetables. In 2018, tomatoes made a significant contribution of approximately 218 billion Indian rupees (INR) to the Indian economy, specifically in India (Mohan et al., 2023). Tomato plants possess exceptional nutritional value because of their well-proportioned composition of minerals, like calcium, zinc, iron, phosphorus, and potassium; vitamins such as B2, B1, B6, A, C, K, E, folic acid, biotin, pantothenic acid, nicotinic acid; and antioxidants including polyphenolic compounds and carotenoids (Sharma et al., 2009; He et al., 2023).

To address the above-mentioned problem, the present study of plant growth-promoting rhizobacteria (PGPR) appears to be a viable route for improving plant resistance in fluoride-stressed conditions. This work investigates the potential of *Pseudomonas aeruginosa* strain PAR as an influential PGPR specifically adapted to handle fluoride-induced stress in tomato (*Lycopersicon esculentum* Mill.) plants. Finally, this investigation adds to our understanding of *P. aeruginosa* strain PAR’s potential as a bio-solution for sustainable agriculture in fluoride-contaminated soils. The findings provide important insights into using PGPR to improve crop resilience and productivity in fluoride-stressed areas.

## 2 Materials and methods

### 2.1 Sample collection and isolation of rhizobacteria

Tomato plant rhizosphere soil was collected from Lakshmangarh (27°48′22.0″N, 75°02′34.4″E), Sikar (Rajasthan), and isolated on YEMA (yeast extract mannitol agar) media purchased from Himedia. The ingredients for the YEMA media are yeast extract (1.0 g L^−1^), dipotassium phosphate (0.5 g L^−1^), mannitol (10.0 g L^−1^), sodium chloride (0.1 g L^−1^), magnesium sulfate (0.2 g L^−1^), Congo red (0.025 g L^−1^), agar (20.0 g L^−1^), and sodium fluoride (5 ppm). It was then incubated at 37°C for 24 h (h), and the recurrent streaking method was used to develop pure cultures. Isolated strains were preserved in 25% glycerol stock solution at −80°C for further use. The isolates that could grow at this concentration of NaF were chosen for the PGP activity assay.

### 2.2 Characterization and identification of PGP activities of rhizobacterial isolates

To assess the colony morphology, isolates were cultured with individual bacterial strains at 37°C for 24 h and examined for margin, color, elevation, and surface morphology under a microscope, and gram staining was done using the standard method. For the selected bacterial strains, slight modifications were made to the following methods to qualitatively estimate their PGP activities.

#### 2.2.1 Catalase estimation

The catalase enzyme formation efficacy of isolated bacteria was checked. Catalase hydrolyzes H_2_O_2_ into H_2_O and O_2_ in bacterial strains. Firstly, smears of isolates were prepared on separate glass slides, and then a few drops of H_2_O_2_ were added to the slide. The production of gas bubbles and effervescence showed a positive test (Kumari et al., 2018).

#### 2.2.2 Estimation of HCN

The determination of HCN production was conducted according to the methodology outlined by Lorck (1948). The isolates were streaked on a nutrient agar plate that had been modified and supplemented with glycine at a concentration of 4.4 grams per liter. Subsequently, Whatman filter paper was immersed in a solution containing sodium carbonate (2%) and picric acid (0.5%) and then placed on the upper surface of the Petri plates. Subsequently, the plates were hermetically sealed with parafilm and placed in an incubator for 4 days at a temperature of 28°C. The presence of hydrogen cyanide (HCN) is indicated by the development of color ranging from orange to red.

#### 2.2.3 Ammonia production

Freshly cultured bacterial strains were separately inoculated in 10 mL of peptone water in test tubes, then they were put in an incubator for 48 h at 28°C. Post incubation, Nessler’s reagent (0.5 mL) was added to each test tube. The appearance of a brown to yellow color indicated the production of ammonia (Dinesh et al., 2015).

#### 2.2.4 Solubilization of phosphate

The isolates were subjected to qualitative measurement of phosphate solubilization using Pikovskaya’s agar. The isolated strains were spread on Pikovskaya’s medium plates and then placed in an incubator at a temperature of 28°C for 5 days. The presence of a halo zone encircling the colony indicates the isolates’ ability to solubilize phosphate (Bhattacharyya et al., 2017).

#### 2.2.5 Indole acetic acid estimation

Indole acetic acid (IAA) production by isolates was checked using the protocol mentioned by Brick et al. (1991). Bacterial cultures were inoculated in a nutrient broth medium supplemented with 1 g L^−1^ of tryptophan. Flasks were incubated under shaking conditions at 80 rpm at 30°C for 4 days. Isolates were then harvested by centrifugation at 4000 rpm for 5 min. Two drops of 10 m-orthophosphoric acid and Salkowski reagent (4.0 mL) were mixed with supernatant (2.0 mL). The mixture was kept at room temperature for incubation. The formation of pink color indicated IAA production.

#### 2.2.6 Siderophore production

The production of siderophores was assessed using a procedure proposed by Alexander and Zuberer (1991). Bacteria were inoculated on chrome Azurol S (CAS) agar plates and incubated at 30°C for 24 h. Post incubation, siderophore formation was confirmed by the change in the color of the media from blue to orange.

#### 2.2.7 Nitrogenase estimation

The use of Jensen’s agar medium facilitated the identification of free aerobic nitrogen fixers in the absence of nitrogen. The Petri plates were sterilized and then filled with medium. The isolates were subjected to centrifugation in physiological saline to remove any residual nitrogen from the preceding media. Each isolate was inoculated, followed by incubation at a temperature of 28 ± 20°C for 2 days. The isolates that showed growth were subsequently streaked on Jensen’s agar media to verify their ability to perform nitrogen fixation (Vishwakarma et al., 2017).

### 2.3 16S rRNA sequence examination and phylogenetic tree generation of bacterial isolates

The chosen isolates were identified using 16S rRNA sequence analysis (Sharma et al., 2023) with minor changes. The genomic DNA was isolated using the phenol-chloroform-isoamyl alcohol (PCI) technique with slight modifications. A nano spectrophotometer (Eppendorf Biospectrometer) was used to assess the quantity and quality of the isolated DNA. The 16S rRNA gene was amplified under standard conditions in a thermal cycler (Applied Biosystems Microamp Optical 96-Well Reaction plate). The PCR products were sequenced bidirectionally using the forward primer GGATGAGCCCGGCCTA and the reverse primer CGGTGTGTACAAGGCCCGG.

In order to examine the evolutionary history of the taxa investigated, the Jukes–Cantor model and maximum likelihood approach were utilized to evaluate the evolutionary history of the studied species. To denote evolutionary history, the bootstrap consensus tree was estimated from 1,000 replicates. For the heuristic search, initial trees were created by implementing the Neighbor–Join and BioNJ algorithms to a matrix of pairwise distances calculated using the Jukes–Cantor model and selecting the topology with the highest log likelihood value. Altogether, there were 1,402 locations in MEGA11, and evolutionary analyses were performed (Jukes and Cantor, 1969).

### 2.4 Effect of NaF on the growth kinetics of selected bacteria

Among all the identified 16 isolates, three potential isolates with PGP activities were selected for the growth kinetic study. The experiment is carried out by a BioTek Eon High-Performance Microplate Spectrophotometer (Rogers et al., 2022). Freshly grown cultures of all three selected isolates were inoculated in nutrient broth amended with 10, 20, 40, and 80 cultures ppm NaF concentrations and incubated at 37°C for 70 h, along with their respective controls.

### 2.5 Growth rate

Jacques Monod’s Monod model was presented in 1942 as an intriguing subject. This model highlights the connection between the substrate utilization rate and the specific growth rate in a bioreactor. It is remarkable how different factors can affect the growth and production of organisms in a controlled environment (Monod, 1949). Managing the growth rate of microorganisms is a crucial aspect of working with fermentation. This helps to maintain a suitable environment and substrate levels that fall within the desired range. As we gain a better understanding of the microbial processes involved, we realize how vital it is to manage the system carefully (Winkelhorst et al., 2023). To estimate a specific growth rate, a model based on cell growth kinetics can be used. In the log phase, the specific growth rate of all three isolates is calculated using the Monod kinetics method.

Monod kinetics (Monod, 1949): *μ* = *d*(*x*)/*X*_0_*dt*; where, *μ* = specific growth rate, *d*(*x*) = biomass of cell produced, *X*_0=_ original biomass of cells, and *dt* = time.


$$\mu =d\left(x\right)/X_{0}dt$$

where, *μ* = specific growth rate, (*x*) = biomass, and *dt* = time.

### 2.6 Seed biopriming with *Pseudomonas aeruginosa* strain PAR

*P. aeruginosa* strain PAR is better suited for fluoride-contaminated areas than the other two *Bacillus* strains studied in the present study as it has an adaptive ability toward fluoride toxicity. Therefore, we selected *P. aeruginosa* strain PAR for further study.

For greenhouse studies, seeds of tomato S-22 (*L. esculentum* Mill.) were purchased and disinfected using 70% ethanol and 0.5% sodium hypochlorite.

To make the *P. aeruginosa* strain PAR inoculum, 24 h grown bacterial culture was added to yeast extract mannitol broth and kept in an incubator for 24 h at 28°C in a shaking condition at 120 rpm. After incubation, the bacterial suspension was subjected to centrifuge to extract bacterial biomass and then suspended in distilled water. The absorbance was recorded at 600 nm and corrected to 0.1, corresponding to 10^7^ CFU mL^−1^. *L. esculentum* Mill. seeds were dipped in bacterial suspension for 30 min. Tomato seeds were submerged in sterile distilled water as a control.

The potting soil was initially dehydrated in the atmosphere to eliminate any moisture. Subsequently, it underwent filtration using a meticulous 2 mm sieve to eliminate contaminants and achieve a uniform texture. Ultimately, the soil that had been filtered was subjected to sterilization in an autoclave to eliminate any potentially detrimental bacteria or organisms that would impede the growth of plants. A plastic bag measuring 25 cm in width and 10 cm in height was filled with 5 kg of soil. Each pot was planted with 10 tomato seeds. The plants were provided with sterile and deionized water on a regular basis to ensure optimal seed germination. The entirety of the cannabis research was conducted in an unregulated greenhouse, with only natural lighting and an ambient temperature.

### 2.7 Pot experiments

The pot study was performed using a completely randomized block design to evaluate the capability of the multi-beneficial NaF-tolerant *P. aeruginosa* strain PAR to lessen the harmful effects of fluoride stress on tomato plants. The pot soil was acquired from the agriculture field of Mody University. The physicochemical properties of experimental soil were examined. Experimental soil with different NaF concentrations, i.e., 10 ppm, 20 ppm, 40 ppm, and 80 ppm, were prepared for fluoride stress conditions. The details of growing conditions and seed treatment are mentioned in Table 1.

**Table 1:** Information on growing conditions and seed treatments for pot trials.

Growing conditions	Seed treatments
Non-NaF (0 ppm)	Non-bacterized seeds (positive control)
NaF 10 ppm	Non-bacterized seeds (negative control)
	*Pseudomonas aeruginosa* strain PAR bacterized seeds
NaF 20 ppm	Non-bacterized seeds (negative control)
	*Pseudomonas aeruginosa* strain PAR bacterized seeds
NaF 40 ppm	Non-bacterized seeds (negative control)
	*Pseudomonas aeruginosa* strain PAR bacterized seeds
NaF 80 ppm	Non-bacterized seeds (negative control)
	*Pseudomonas aeruginosa* strain PAR bacterized seeds

### 2.8 Seedling growth

The pot experiment was meticulously structured to achieve the optimal growing conditions for the 20 plants cultivated for each treatment. In order to do this, two well-established seedlings were introduced into each pot, while the remaining seedlings were eliminated as they developed. During the 60 days period of the experiment, the morphological characteristics of each seedling were carefully observed to collect valuable information. The percentage of seed germination was recorded up to 1 month following seeding. The shoot height was determined using a scale, from the plant’s tip to the stem’s end. The length of the root was measured from the collar region to the end of the root using a scale. The fresh weight of the roots and shoots was measured after harvest. After 5 days of drying at 40°C in a hot air oven, when a constant weight was achieved, the root and shoot dry weight was recorded using a weighing machine. The leaf area was marked out on graph paper. The relative water content was estimated by following the procedure mentioned by Teulat et al. (2003) and the relative water content RWC (%) was calculated using the following equation:


$$\text{RWC}\left(\%\right)=\frac{\text{Fresh}\;\text{weight}-\text{Dry}\;\text{weight}}{\text{Fully}\;\text{turgid}\;\text{weight}-\text{Dry}\;\text{weight}}\times 100$$

### 2.9 Biochemical parameters

The phenol-sulfuric acid method was utilized to measure total soluble sugar content (Krishnaveni et al., 1984). The proline and glycine betaine were estimated using the procedure mentioned by Grattan (1999) and Patel et al. (2014), respectively. The content of total chlorophyll was measured using the method proposed by Arnon (1949). The content of malondialdehyde (MDA) was estimated to determine lipid peroxidation (Heath and Packer, 1968). Antioxidant enzymes like catalase (CAT), superoxide dismutase (SOD), glutathione reductase (GR), and ascorbate peroxidase (APX) were measured following the protocol proposed by Nakano and Asada (1981), Aebi (1984), Beyer and Fridovich (1987), and Smith et al. (1988), respectively. The study performed a mineral analysis of tomato plant leaves, with a specific emphasis on Calcium, Iron, Potassium, Magnesium, and Sodium. The analysis employed approaches derived from the methodology outlined by Piper (1944), Johnson and Ulrich (1959), and Kacar and Inal (2008). The F^−^ concentration in soil and plants was measured using an ion selective analyzer (Mishra et al., 2014).

### 2.10 Statistical analysis

The principal component analysis (PCA) was done to estimate noteworthy associations between the measured parameters. Pearson’s correlation coefficient analysis was done to determine the associations among various physicochemical parameters. To discover important variations between comparison means, similarity groups of all measured parameters were performed using a one-way ANOVA Tukey’s Multiple range test.

## 3 Results

### 3.1 Isolation and PGP activities of isolated rhizobacteria

Pure cultures of PGPRs were developed from the samples collected, and 16 isolates were screened for their properties as per Table 2. The results demonstrated that isolates 1–13 have a maximum and 14–16 numbered isolates demonstrated only three different types of PGP activities. Furthermore, isolates number 7, 11, and 12 demonstrated six out of seven major and intense analyzed activities. Isolates 11 and 12 have not given HCN production activity, and isolate 7 showed negative results for PO4 solubilization capacity. Therefore, these three (7, 11, and 12) isolates were subjected to further studies.

**Table 2:** Displays the data about the primary and secondary screening of the isolates.

No.	Primary screening		Secondary screening						
	Gram +/−	Colony morphology	NH_4_	HCN	Sidero-phore	IAA	PO_4_	Catalase	Nitrogenase
1.	+	Convex shape, flat, off-white	−	−	++	++	+	+	+
2.	+	Oval shape, flat, pinkish color	++	++	−	++	+	+	+
3.	+	Circular shape, flat, white color	+	++	−	++	+	+	+
4.	+	Oval shape, flat, pinkish color	+	−	+	−	+	+	+
5.	+	Flat, irregular edges, white color	−	+	++	−	−	+	+
6.	+	Opaque, rough, white color	+	−	−	++	++	+	+
7.	+	Smooth, circular, flat, and off-white	++	++	++	++	−	+	++
8.	+	Flat, irregular edges, white color	+	++	−	++	−	+	+
9.	+	Rough, opaque, white color	++	+	−	−	+	+	+
10.	+	Rough, opaque, white color	−	−	++	++	+	+	+
11.	−	Small, smooth, flat edges.	++	−	++	++	++	+	++
12.	+	Oval shape, flat, pinkish color	++	−	++	++	++	+	++
13.	+	Circular, flat, gray color	++	−	−	−	−	+	+
14.	−	Oval, irregular edges, off-white color	++	−	−	−	−	+	+
15.	+	Round, flat, pinkish color	++	−	−		−	+	+
16.	+	Round, convex, pinkish color	−	−	++	−	−	+	+

Gram staining, analysis of colony shape, and qualitative assessment of PGP (plant growth promoting) activities were conducted on all 16 strains that were isolated from the high fluoride region of Rajasthan (27° 48′22.0″N, 75° 02′ 34.4″E). ^+^Moderate level of PGP activity; ^++^High level of PGP activity.

### 3.2 The effect of NaF on bacterial growth

**Figure 1:**
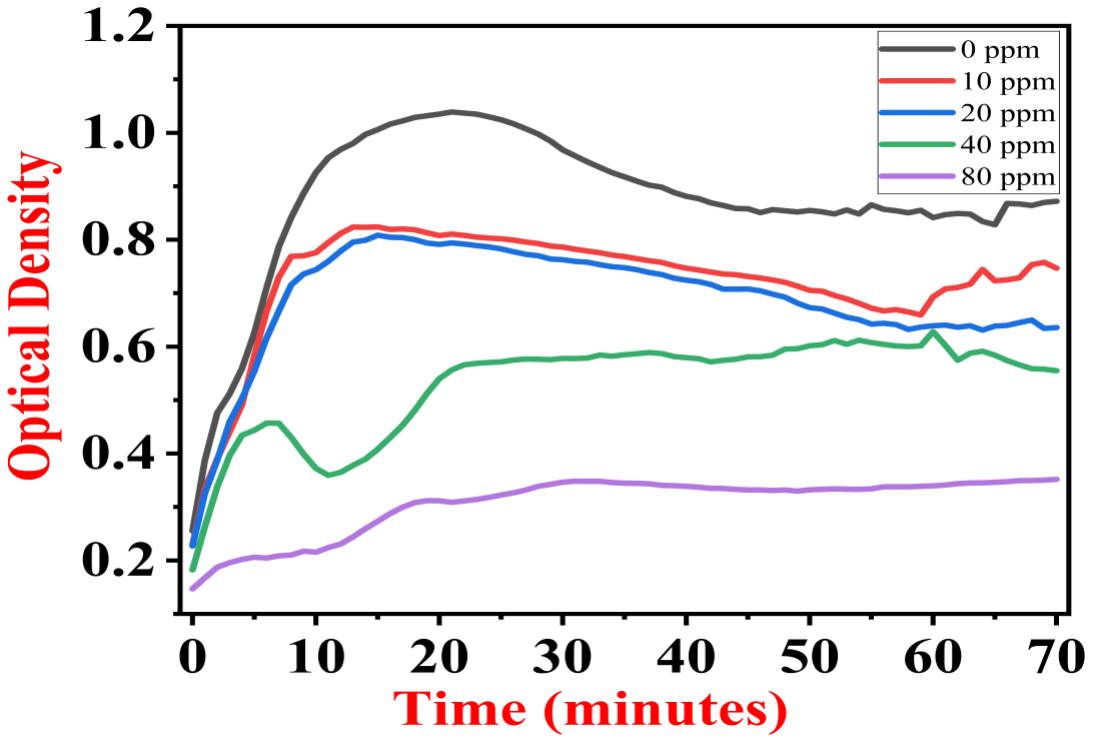
Growth graph of isolate S1 (7) in the existence of 10, 20, 40, and 80 ppm NaF conc. and 0 as a positive control.

**Figure 2:**
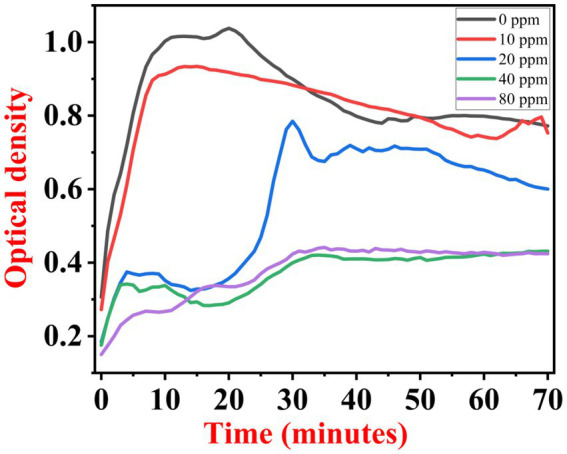
Growth graph of isolate S3 (12) in the existence of 10, 20, 40, and 80 ppm NaF conc. and 0 as a positive control.

**Figure 3:**
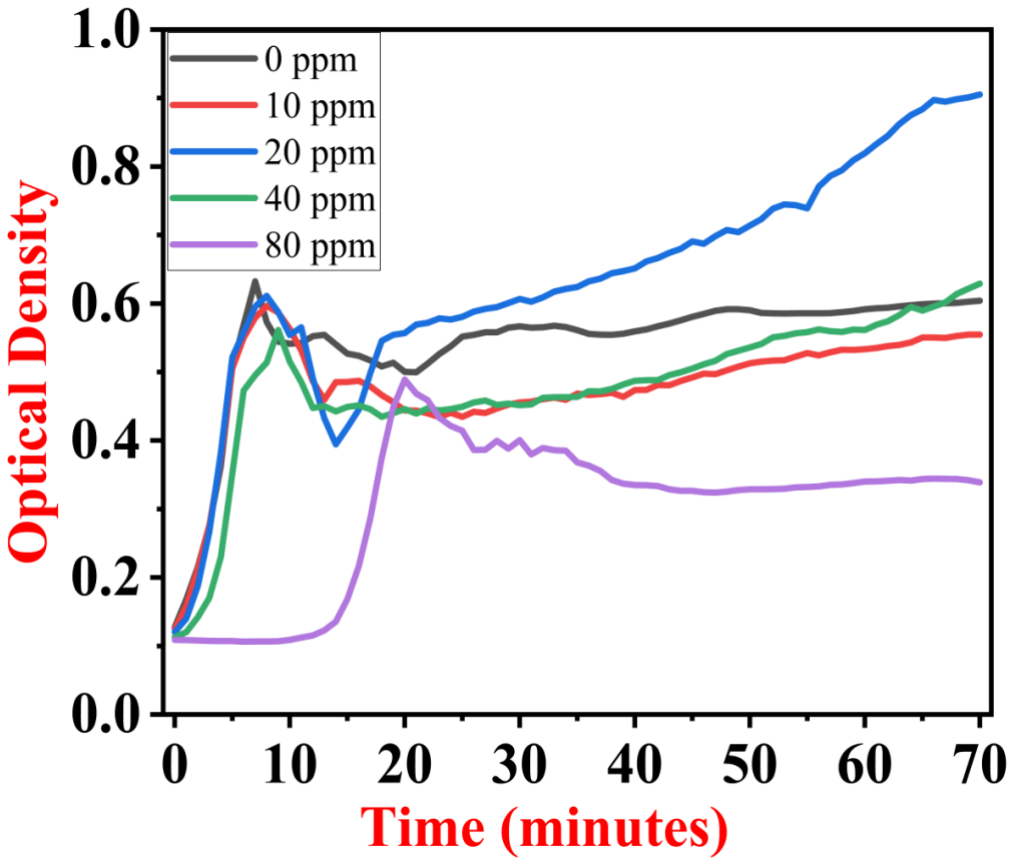
Growth graph of isolate S2 (11) in the existence of 10, 20, 40, and 80 ppm NaF conc. and 0 as a positive control.

The growth kinetics study was carried out using a BioTek Eon High-Performance Microplate Spectrophotometer. Approximately 2 μL freshly grown culture of all three [(S1) 7, (S2) 11, and (S3) 12] selected isolates were inoculated in 198 μL nutrient broth amended with 10 ppm, 20 ppm, 40 ppm, and 80 ppm of NaF concentrations for 72 h. The results of all three strains are demonstrated as per the respective graphs 1–3. The demonstrated observations show that both *Bacillus* species (S1 and S3) showed reduced growth in the presence of fluoride, especially at higher (40 and 80) ppm (Figures 1, 2). At 10 ppm fluoride toxicity, the reduction in growth was low compared to higher concentrations in all three isolates (Figures 1–3). Interestingly, S1 demonstrates almost similar growth in the case of 10, 20, and 40 ppm exposure during the initial hours of incubation. The S2 isolate showed adaptation against fluoride toxicity at 20 ppm and, therefore, growth was even better than in the control (Figure 3). Even at higher concentrations, the S2 isolates demonstrated adaptation after 16 h of incubation and thereafter exhibited improved growth in comparison to the other two isolates. The S3 isolate was only suitable for the 10 ppm fluoride toxicity level; when exposed to higher (20, 40, 80) ppm, growth reduced significantly (Figure 2).

### 3.3 Specific growth rate

The presented data shows that the growth rate of S1 isolate is 0.081 h^−1^ without NaF. Interestingly, the growth rate slightly increased to 0.085 h^−1^ with the presence of 10 ppm NaF. However, it decreased with 20 ppm concentration. The growth rate of the S2 isolate was 0.059 h^−1^ without NaF. It increased to 0.062 h^−1^ with the presence of 10 ppm NaF. Significantly, it increased to 0.073 h^−1^ with 20 ppm NaF. Meanwhile, the S3 isolate had a growth rate of 0.035 h^−1^ without fluoride. In the presence of 10 ppm NaF, the rate increased to 0.040 h^−1^. Nevertheless, the rate started to decrease at 20 ppm concentration. With an increase in the NaF concentration from 10 ppm to 20 ppm, it was noticed that S2 showed the maximum specific growth rate. However, the growth rates of S1 and S3 isolates were comparatively slower than that of S2 (Table 3).

**Table 3:** Specific growth rate of three isolates of rhizobacterial species.

Isolate	Code	NaF conc. (ppm)	Specific growth rate at log phase (12 h)
S1	7	0	0.081 h^−1^
		10	0.085 h^−1^
		20	0.069 h^−1^
S2	11	0	0.059 h^−1^
		10	0.062 h^−1^
		20	0.073 h^−1^
S3	12	0	0.035 h^−1^
		10	0.040 h^−1^
		20	0.036 h^−1^

### 3.4 Molecular identification and phylogenetic analysis of the isolates

The 16S rRNA analysis of isolate numbers S1 (7), S2 (11), and S3 (12) results in the identification of the strains as per Table 4. The genomic sequence of all three rhizobacterial isolates has been deposited to the NCBI GenBank, and their accession numbers are listed in Table 4.

**Table 4:** Results of 16S rRNA sequencing and species identification of isolates.

Isolate number	Accession No.	Species Identified
(S1) 7	OR427951	*Bacillus* sp. *Marseille* P3606
(S2) 11	OR427950	*Pseudomonas aeruginosa* PAR
(S3) 12	OR427949	*Bacillus cereus* S8

**Figure 4:**
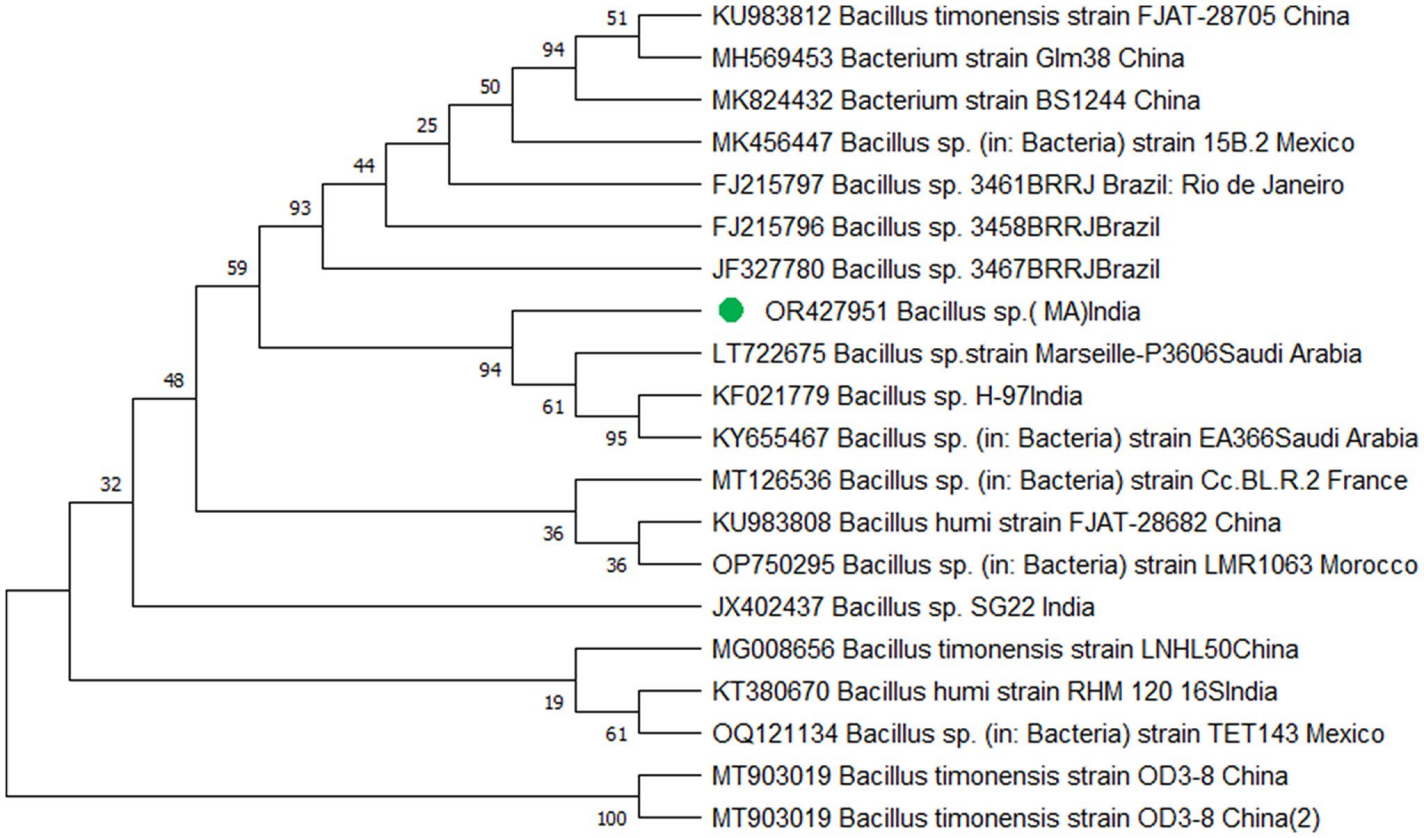
Phylogenetic tree of isolate no. (S1) 7 *Bacillus* sp. *Marseille* P3606.

**Figure 5:**
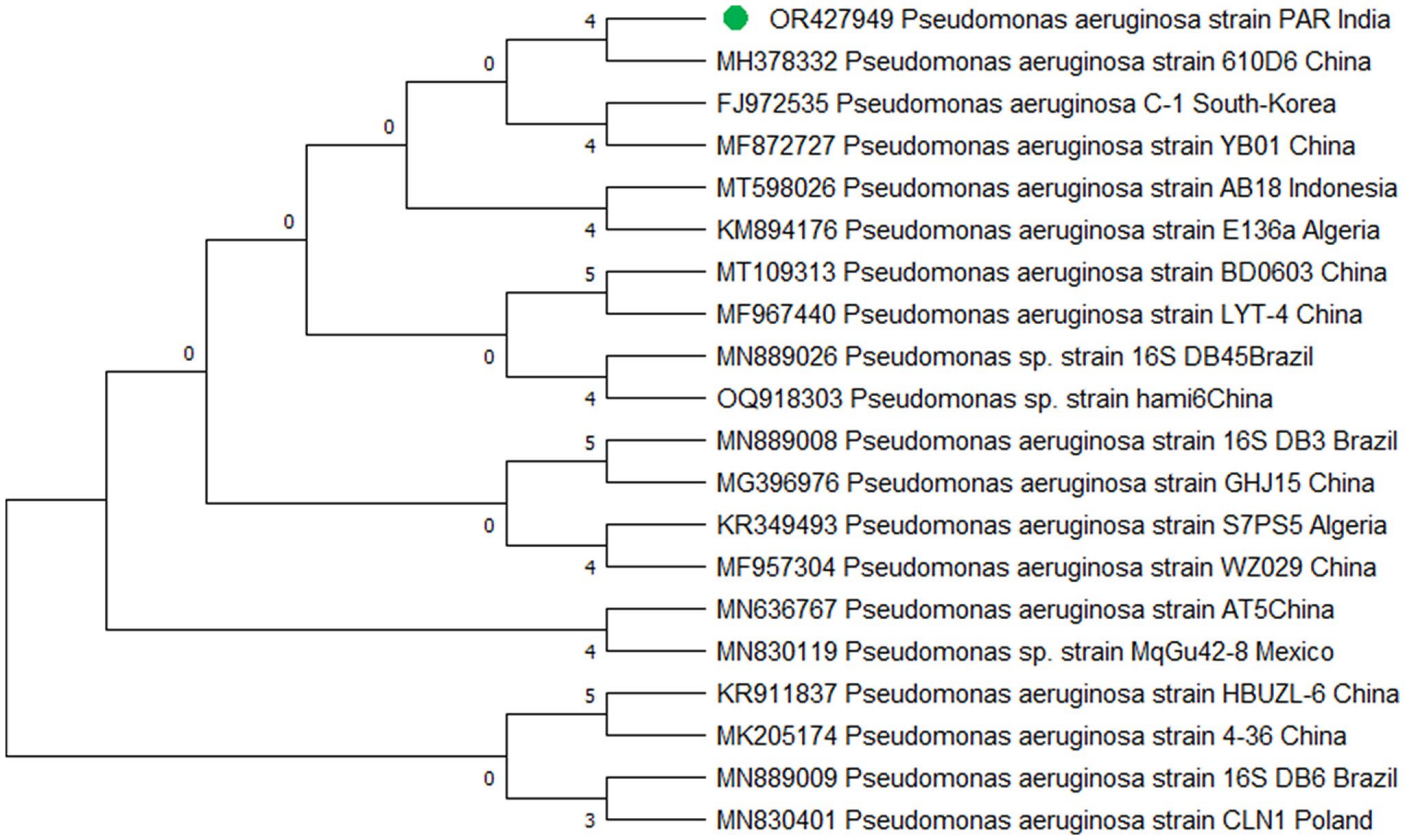
Phylogenetic tree of isolate no. (S2) 11 *Pseudomonas aeruginosa* PAR.

**Figure 6:**
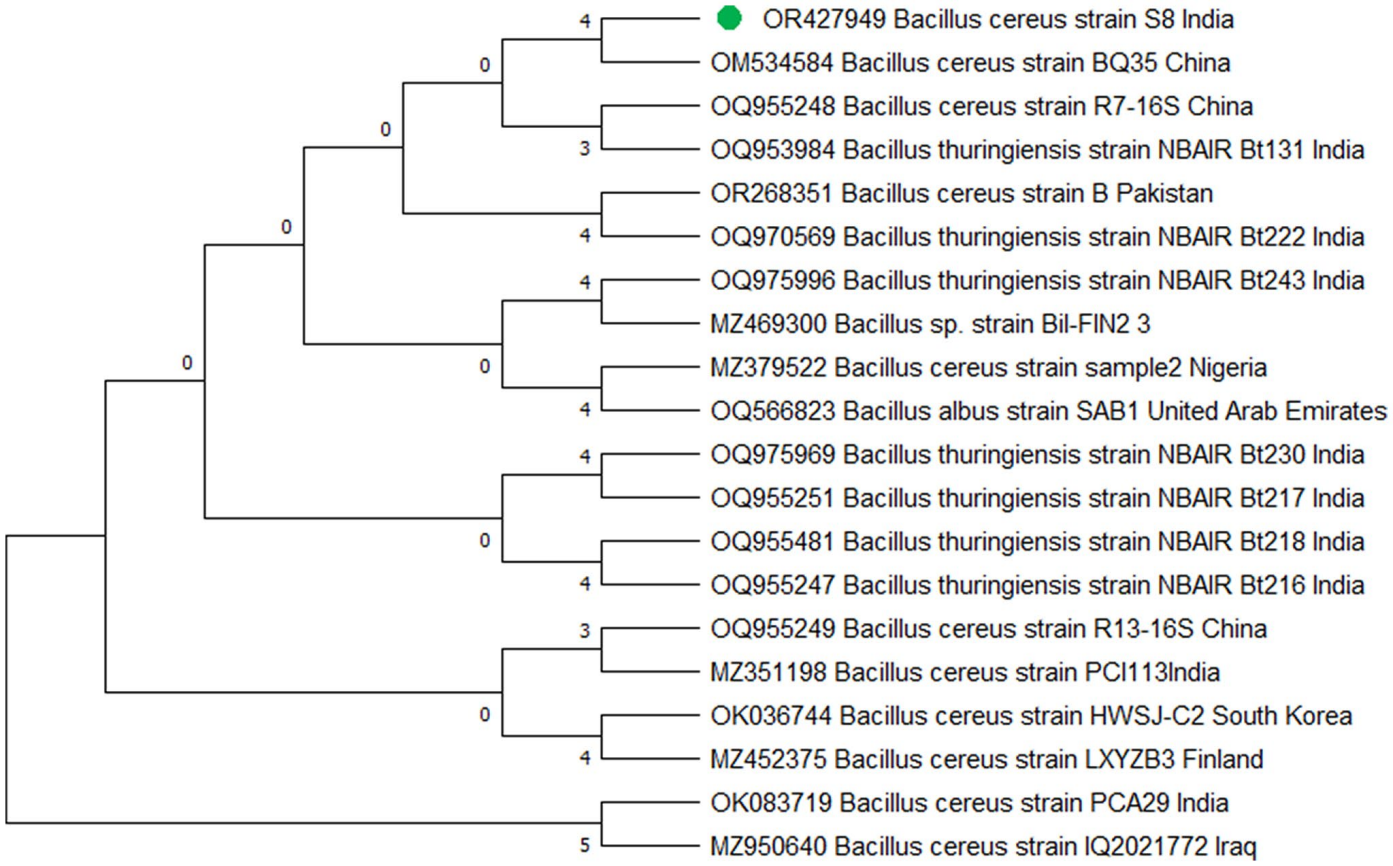
Phylogenetic tree of isolate no. (S3) 12 *Bacillus cereus* S8.

The study’s findings revealed that isolate S1 (7) is closely related (100%) to the *Bacillus Marseille* P306 strain and belongs to the *Bacillus* genus. It was identified as *Bacillus* sp. (MA) (Figure 4). Isolate S2 (11) was found to have a high similarity (99%) to *P. aeruginosa* strain 610D6 and belongs to the *Pseudomonas* genus. It was named *P. aeruginosa* strain PAR (Figure 5). Lastly, isolate S3 (12) was discovered to be closely related (100%) to the *Bacillus cereus* BQ35 strain; it belongs to the genus *Bacillus* and was named *B. cereus* strain S8 (Figure 6).

### 3.5 Physiological determination of plant growth parameters

The impact of various concentrations of sodium fluoride (NaF) on the percentage of seed germination, relative water content, root length, shoot height, leaf area, and shoot-root dry and fresh weight were measured in sodium fluoride (NaF) conditions and in non-sodium fluoride (NaF) conditions as a control. The seed germination results revealed that bio-inoculation of *P. aeruginosa* strain PAR expressively enhanced the germination (%) of tomato plants compared to NaF + non-inoculated (non-bacterized seeds) plants. The germination rate of non-NaF seedlings without inoculation was 82% under NaF stress conditions. This rate decreased to 56% under 10ppm NaF stress, 47% under 20ppm NaF stress, 39% under 40ppm NaF stress, and 21% under 80ppm NaF stress. The seedlings treated with *P. aeruginosa* strain PAR showed a germination rate of 79% under 10 ppm NaF stress, 66% under 20 ppm NaF stress, 51% under 40 ppm NaF stress, and 34% under 80 ppm NaF stress (Table 5).

**Table 5:** Effects of tomato seed bio-priming with *Pseudomonas aeruginosa* strain PAR on the plant growth parameters under sodium fluoride stressed conditions.

NaF concentration (ppm)	Treatment	Shoot height (cm)	Root length (cm)	Leaf area (cm^2^)	Germination (%)
0 ppm	Positive control	48.76 ± 2.5d	14.44 ± 1.8ef	29 ± 2.3f	82 ± 7.1d
10 ppm	NaF + non-bacterized seeds	39.9 ± 2.5d	12.68 ± 1.3e	19 ± 1.7de	56 ± 5.1c
	NaF + bacterized seeds	59.12 ± 4.1e	16.89 ± 1.3f	27.3 ± 2.5f	79 ± 5.7d
20 ppm	NaF + non-bacterized seeds	32.76 ± 1.9c	10.14 ± 0.7d	16 ± 1.5d	47 ± 3.9c
	NaF + bacterized seeds	43.12 ± 3.2d	13.12 ± 0.8e	21.36 ± 1.5e	66 ± 5.1dc
40 ppm	NaF + non-bacterized seeds	24.21 ± 1.1b	7.34 ± 0.5c	11 ± 0.9c	39 ± 2.9b
	NaF + bacterized seeds	33.56 ± 2.3c	10.32 ± 0.8d	13.99 ± 0.7d	51 ± 3.2c
80 ppm	NaF + non-bacterized seeds	11.24 ± 0.9a	4.18 ± 0.2a	7 ± 0.6a	21 ± 1.1a
	NaF + bacterized seeds	13.12 ± 1.2a	5.11 ± 0.3b	8.74 ± 0.4b	34 ± 2.2b

Under 10 ppm NaF, 20 ppm NaF, 40 ppm NaF, and 80 ppm NaF stress conditions, *P. aeruginosa* strain PAR considerably enhanced the root length, shoot height, leaf area, shoot–root dry and fresh weight, and relative water content compared to non-inoculated tomato plants. In the non-inoculated 10 ppm NaF, 20 ppm NaF, 40 ppm NaF, and 80 ppm NaF stress conditions, there was a noteworthy fall in root length, shoot height, leaf area, shoot–root dry and fresh weight, and relative water content of tomato plants (Table 6).

**Table 6:** The effect of *Pseudomonas aeruginosa* strain PAR seed biopriming on the fresh weight of the root and shoot, dry weight of the shoot and root, and relative water content of tomato plants is examined under varying concentrations of sodium fluoride.

NaF concentration (ppm)	Treatment	Shoot fresh weight (gm)	Shoot dry weight (gm)	Root fresh weight (gm)	Root dry weight (gm)	RWC (%)
0 ppm	Positive control	10.58 ± 1.1f	4.44 ± 0.39d	0.67 ± 0.06ef	0.27 ± 0.02d	81.2 ± 5.2e
10 ppm	NaF + non-bacterized seeds	6.74 ± 0.62d	3.05 ± 0. 24c	0.55 ± 0.04d	0.21 ± 0.01c	73.3 ± 4.9de
	NaF + bacterized seeds	10.35 ± 0.8ef	4.42 ± 0.54d	0.79 ± 0.06f	0.31 ± 0.02d	84.7 ± 6.1e
20 ppm	NaF + non-bacterized seeds	4.74 ± 0.4c	2.78 ± 0.23c	0.42 ± 0.03c	0.17 ± 0.01b	59.1 ± 3.4c
	NaF + bacterized seeds	8.89 ± 0.7e	3.12 ± 0.27c	0.65 ± 0.05e	0.27 ± 0.002d	68.9 ± 4.2d
40 ppm	NaF + non-bacterized seeds	3.21 ± 0.42b	2.12 ± 0.19b	0.31 ± 0.02b	0.15 ± 0.01b	47.3 ± 3.1b
	NaF + bacterized seeds	6.74 ± 0.6d	2.97 ± 0.22c	0.52 ± 0.03d	0.21 ± 0.02c	55.9 ± 3.0c
80 ppm	NaF + non-bacterized seeds	2.45 ± 0.2a	1.23 ± 0.12a	0.19 ± 0.01a	0.11 ± 0.02a	33.1 ± 2.1a
	NaF + bacterized seeds	4.12 ± 0.3c	2.17 ± 0.23b	0.36 ± 0.03bc	0.16 ± 0.01b	42.1 ± 2.7b

### 3.6 Soluble sugar, glycine betaine, and proline

**Figure 7:**
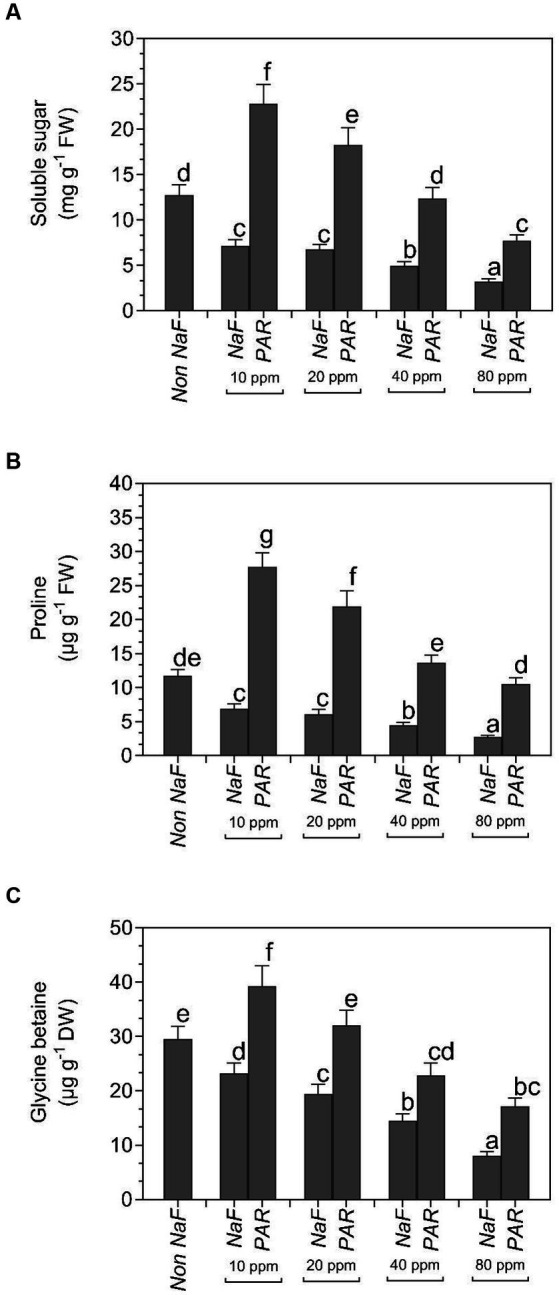
Effects of *Pseudomonas aeruginosa* strain PAR seed biopriming on the **(A)** soluble sugar **(B)** proline **(C)** glycine betaine leaf extracts of tomato plants under different concentrations of NaF. Data were investigated using a one-way ANOVA Tukey’s multiple concentrations range test (*p* < 0.05). Diverse small letters have substantial differences.

The *P. aeruginosa* strain accumulated the most soluble sugar content in 10 ppm (22.87 mg g^−1^ FW), 20 ppm (18.32 mg g^−1^ FW), 40 ppm (12.4 mg g^−1^ FW), and 80 ppm (7.74 mg g^−1^ FW) NaF stress conditions. Under 10 ppm NaF, 20 ppm NaF, 40 ppm NaF, and 80 ppm NaF stress conditions, uninoculated plants showed 7.17 mg g^−1^ FW, 6.78 mg g^−1^ FW, 4.99 mg g^−1^ FW, and 3.21 mg g^−1^ FW soluble sugar, respectively. The non-NaF plant accumulated 12.78 mg g^−1^ FW of soluble sugars (Figure 7A).

Proline accumulation was greatest in *P. aeruginosa* strain PAR at 10 ppm (27.73 μg g^−1^ FW), 20 ppm (21.9 μg g^−1^ FW), 40 ppm (13.7 μg g^−1^ FW), and 80 ppm (10.5 μg g^−1^ FW) NaF stress conditions. Under 10 ppm NaF, 20 ppm NaF, 40 ppm NaF, and 80 ppm NaF stress conditions, uninoculated plants showed 6.89 μg g^−1^ FW, 6.12 μg g^−1^ FW, 4.43 μg g^−1^ FW, and 2.78 μg g^−1^ FW proline content, respectively. Proline accumulation was found to be 11.75 μg g^−1^ FW in non-NaF plants (Figure 7B).

The highest buildup of glycine betaine (GB) was found in *P. aeruginosa* strain PAR at 10 ppm (39.3 μg g^−1^ DW), 20 ppm (32.1 μg g^−1^ DW), 40 ppm (22.9 μg g^−1^ DW), and 80 ppm (17.2 μg g^−1^ DW) NaF stress conditions. Under 10 ppm NaF, 20 ppm NaF, 40 ppm NaF, and 80 ppm NaF stress conditions, uninoculated plants showed 23.2 μg g^−1^ DW, 19.4 μg g^−1^ DW, 14.5 μg g^−1^ DW, and 8.12 μg g^−1^ DW GB, respectively. The buildup of osmolyte GB in the non-NaF plant was 29.5 μg g^−1^ DW (Figure 7C).

### 3.7 Chlorophyll, MDA, and antioxidant

**Figure 8:**
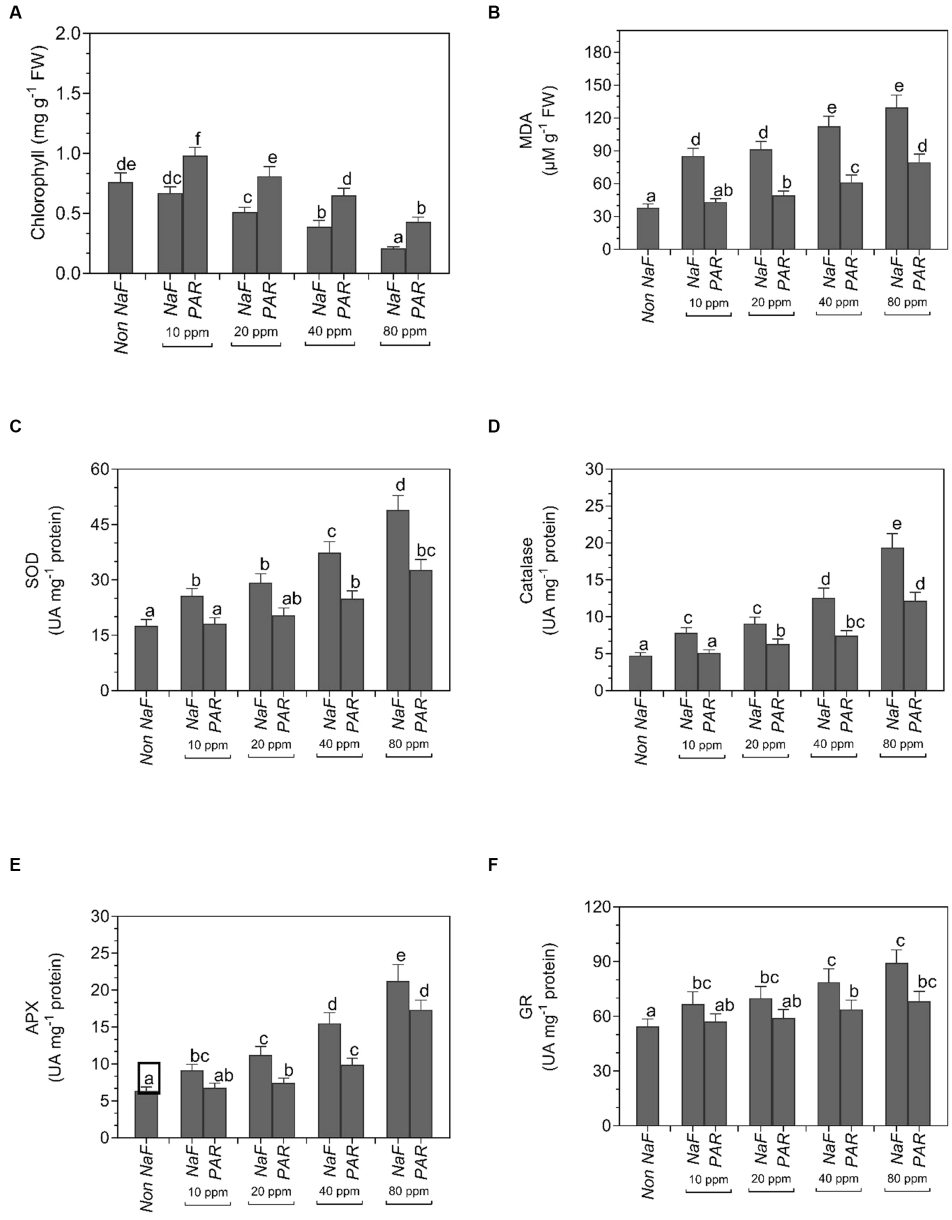
Effects of *Pseudomonas aeruginosa* strain PAR seed biopriming on the **(A)** chlorophyll, **(B)** MDA, **(C)** superoxide dismutase, (SOD) activity, **(D)** catalase activity, **(E)** ascorbate peroxidase (APX) activity, and **(F)** glutathione reductase (GR) activity of leaf extracts of tomato plants under different concentration of sodium fluoride. Data were investigated using a one-way ANOVA Tukey’s multiple range test (*p* < 0.05). Diverse small letters have substantial differences.

The chlorophyll content decreased in all four uninoculated NaF circumstances (10 ppm, 20 ppm, 40 ppm, and 80 ppm) in comparison to both NaF-treated and non-NaF plants. Nevertheless, plants treated with *P. aeruginosa* strain PAR exhibited a noteworthy augmentation in the overall amount of chlorophyll. Under NaF stress conditions, the chlorophyll concentration was 0.98 mg g^−1^ FW at 10 ppm, 0.81 mg g^−1^ FW at 20 ppm, 0.65 mg g^−1^ FW at 40 ppm, and 0.43 mg g^−1^ FW at 80 ppm. Conversely, the plant without NaF had a total chlorophyll content of 0.76 mg g^−1^ FW (Figure 8A).

Under 10 ppm NaF, 20 ppm NaF, 40 ppm NaF, and 80 ppm NaF stress conditions, MDA was significantly decreased in comparison with non-inoculated plants. Non-inoculated tomato plants had the maximum MDA in 10 ppm (85.23 μM g^−1^ FW), 20 ppm (91.6 μM g^−1^ FW), 40 ppm (112.5 μM g^−1^ FW), and 80 ppm (129.8 μM g^−1^ FW) NaF stress conditions. The MDA of the non-NaF plant was 38.15 μM g^−1^ FW (Figure 8B).

Under 10 ppm NaF, 20 ppm NaF, 40 ppm NaF, and 80 ppm NaF stress conditions, antioxidative enzymes like catalase (CAT), ascorbate peroxidase (APX), superoxide dismutase (SOD), and glutathione reductase (GR) exhibited a noteworthy decline in activity in comparison to non-inoculated tomato plants. Non-inoculated tomato plants showed the maximum activity of SOD in 10 ppm (25.74 UA mg^−1^ protein), 20 ppm (29.23 UA mg^−1^ protein), 40 ppm (37.43 UA mg^−1^ protein), and 80 ppm (48.95 UA mg^−1^ protein) NaF stress conditions. Superoxide dismutase activity in the non-NaF plant was observed to be 17.56 UA mg^−1^ protein (Figure 8C).

The maximum catalase enzyme activity was found in uninoculated 10 ppm (7.85 UA mg^−1^ protein), 20 ppm (9.12 UA mg^−1^ protein), 40 ppm (12.58 UA mg^−1^ protein), and 80 ppm (19.40 UA mg^−1^ protein) NaF stress conditions. The non-NaF plant showed 4.75 UA mg^−1^ protein catalase activity (Figure 8D).

The maximum ascorbate peroxidase enzyme activity was found in uninoculated 10 ppm (9.15 UA mg^−1^ protein), 20 ppm (11.22 UA mg^−1^ protein), 40 ppm (15.52 UA mg^−1^ protein), and 80 ppm (21.23 UA mg^−1^ protein) NaF stress conditions. The APX activity of the non-NaF plant was 6.37 UA mg^−1^ protein (Figure 8E).

Uninoculated 10 ppm (66.8 UA mg^−1^ protein), 20 ppm (69.94 UA mg^−1^ protein), 40 ppm (78.45 UA mg^−1^ protein), and 80 ppm (89.34 UA mg^−1^ protein) NaF-stressed plants exhibited the maximum activity of GR enzyme. The glutathione reductase activity of the non-NaF tomato plant was 54.4 UA mg^−1^ protein (Figure 8F).

### 3.8 Mineral analysis

**Figure 9:**
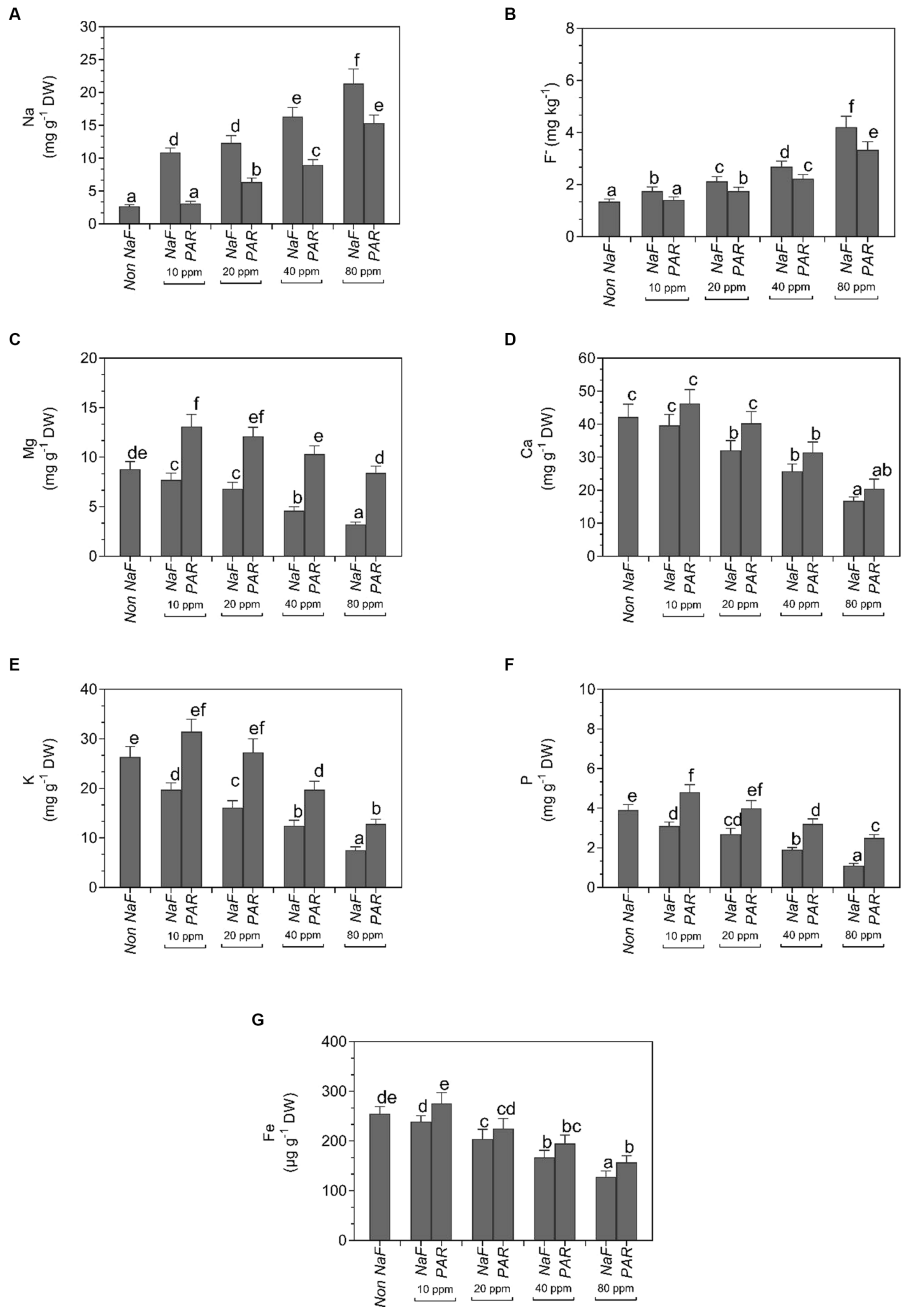
Effects of *Pseudomonas aeruginosa* strain PAR seed biopriming on the **(A)** Na, **(B)** F^−^, **(C)** Mg, **(D)** Ca, **(E)** K, **(F)** P, and **(G)** Fe in tomato leaf extracts under different concentrations of sodium fluoride. Data were investigated using a one-way ANOVA Tukey’s multiple range test (*p* < 0.05). Diverse small letters have substantial differences.

A noteworthy fall in the NaF content of tomato plants was observed when inoculated with *P. aeruginosa* strain at different NaF concentrations (10 ppm, 20 ppm, 40 ppm, and 80 ppm). There was a substantial upsurge in the Mg, K, P, and Fe content of tomato plants when treated with *P. aeruginosa* strain at different NaF concentrations. There was no significant impact on the Ca quantity of tomato leaves when treated with *P. aeruginosa* strain at different NaF concentrations (Figure 9).

### 3.9 Statistical investigation of plant physicochemical and growth parameters

**Figure 10:**
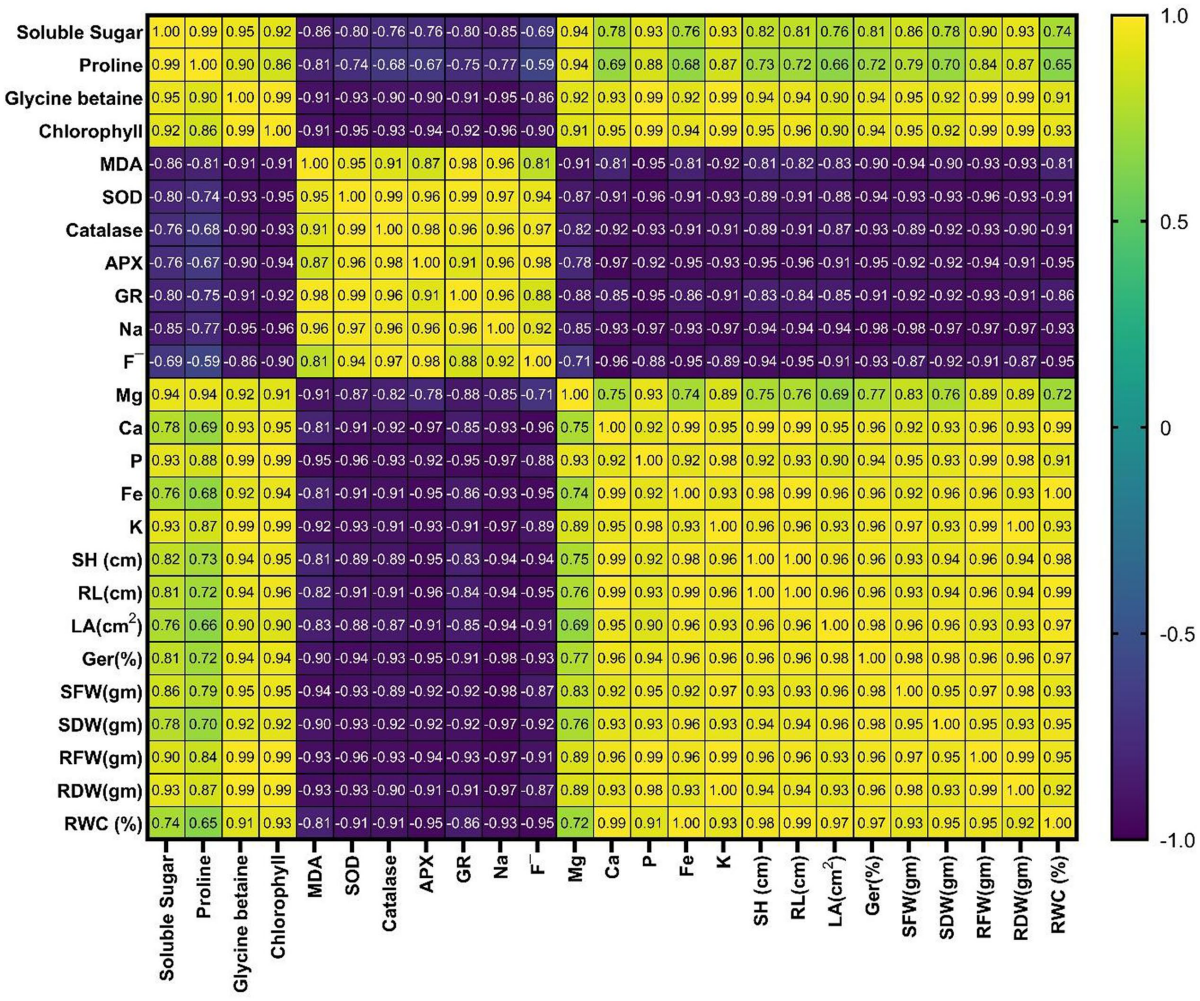
Correlation matrix among the distinctive parameters of tomato plants affected by NaF stress conditions and *Pseudomonas aeruginosa* strain PAR inoculation. MDA, malondialdehyde; GR, glutathione reductase; SOD, superoxide dismutase; APX, ascorbate peroxidase; K, potassium; F^−^, fluoride; Na, sodium; Mg, magnesium; Ca, calcium; P, phosphorus; Fe, iron; RL, root length; SH, shoot height; LA, leaf area; SDW, shoot dry weight; SFW, shoot fresh weight; Ger (%), germination (%); RDW, root dry weight; RFW, root fresh weight; RFA, relative water content.

The diverse oxidative stress indicator metrics are negatively connected with biomass, growth, and biomass parameters of tomato plants (El Moukhtari et al., 2020). There was a positive association of relative water content and percentage of seed germination proportion with Fe and K amount but an adverse association of seed germination (%) proportion with MDA and F-amount, which indicates that K and Fe enhanced the seed germination (%) of tomato plants under stress conditions, while MDA and F^−^ constrained the seed germination (%) of tomato plants under stress conditions. There was an adverse connection between K and Fe content and MDA, Na, and F^−^ amount, which indicates that they reduce the influence of these oxidative parameters in inoculated tomato plants. There was an adverse connection between root length, leaf area, and shoot height and the fluoride amount, which hindered the tomato plant growth under stress conditions. However, there was a positive relationship between shoot height, leaf area, and root length and P, Mg, K, Ca, Fe, and PE content and chlorophyll, which indicated that there was outstanding growth in inoculated tomato plants due to the upsurge in these (P, Mg, K, Ca, Fe, and P content and chlorophyll) physicochemical properties (Figure 10 and Supplementary Tables S1, S2). Therefore, tomato plant biomass and growth parameters are disturbed by NaF stress.

The principal component analysis successfully captured all the fluctuations in the data, as demonstrated by the complete coverage of eigenvalues reaching 100%. The analysis categorized the results into eight categories based on significant differences in physicochemical and growth factors. Group 1 consisted of control tomato plants that differed from inoculated plants by 91.13%, whereas the inoculated tomato plants varied within a range of 4.97% to 0.04% (Supplementary Table S3).

## 4 Discussion

The current study’s findings give useful insights into the impacts of sodium fluoride (NaF) on numerous parameters associated with the growth and development of tomato plants and the potential ameliorative role of *P. aeruginosa* strain PAR in reducing NaF-induced stress. In order to survive in harsh environments, microorganisms possess the ability to endure various pollutants. One such global concern, particularly in India, is fluoride contamination. Industrial activities and synthetic fertilizers are the primary causes of fluoride compound contamination in the environment (Muthu et al., 2023). This environmental risk may endanger plants and other organisms. It is a protoplasmic toxin that can affect the metabolic processes of a variety of microorganisms (Pal et al., 2022). Three bacterial strains were identified in an area with up to 15.0 ppm fluoride concentration (Karanjac, 1993; Choubisa, 2018). These rhizobacterial strains may be capable of actively absorbing fluoride in a liquid medium and can tolerate high NaF fluoride concentrations. In general, PGPRs have many types of mechanisms to deal with harmful substances. With regard to fluoride, PGPRs have a unique class of channel proteins known as putative F-transporters, which they use to reduce the toxicity of fluoride on bacterial cells. These genes are actually riboswitches, a unique class of metabolite-binding RNA structure that is activated by high F concentrations (Chellaiah et al., 2021). It was discovered in this investigation that these isolated PGPR strains could survive high fluoride concentrations.

It seems that there are various mechanisms that enable bacterial strains to tolerate fluoride, such as efflux pumps, intracellular sequestration, enzyme changes, and detoxifying enzymes. In addition, there are increased DNA repair mechanisms, expression of fluoride riboswitches, ion antiporters or transporters, and genetic mutations that contribute to fluoride tolerance. These findings are intriguing and deliver esteemed visions into the mechanisms behind bacterial fluoride tolerance (Brussock and Kral, 1987; Liao et al., 2015; Mukherjee et al., 2015; Haritha and Naphade-Mahajan, 2016; Liao et al., 2017; Mukherjee et al., 2018; Johnston and Strobel, 2019; Katiyar et al., 2020; Chellaiah et al., 2021).

Recent research suggests that certain microbes have evolved a unique mechanism to effectively transport fluoride, which enables them to thrive in fluoride-rich environments. Moreover, these microbes seem to possess the ability to identify specific targets for fluoride, which could have significant implications for comprehending their survival strategies in such harsh conditions.

The present study has compared growth kinetics and specific growth rates to identify potential fluoride-tolerant rhizobacterial isolates. It has been revealed that the particular growth rate is critical to control throughout fermentation because it characterizes the dynamic behavior of microbes. Using a certain growth rate as a control parameter, it could be presumed that the substrate exists in adequate quantities, which may indirectly help control the extracellular environment (Winkelhorst et al., 2023).

There have been various studies conducted to develop fluoride-tolerant microorganisms. According to Mukherjee et al. (2015), an examination of a fluoride-resistant bacteria *Acinetobacter* sp. Rh5 displays defluoridation capabilities; it exhibits a major bioremediation function by concentrating the fluoride anions so that they are less accessible. In light of this, these fluoride-resistant bacteria could be a useful strain for the defluoridation of water in fluoride-contaminated sites. Chellaiah et al. (2021) found *Aeromonas caviae bacteria* 31, 32, and 34 to be fluoride-resistant isolates, along with *Enterobacter cloacae* strain 3, *E. hormaechei* strain 14, *Enterobacter* sp. bacteria 21, and *E. coli bacteria* S2-9, and *E. hormaechei* bacteria 22. Using gene-specific primers, the Fluoride-resistant isolates’ ‘crcB’ gene was greatly amplified.

According to the findings of this study, it appears that exposure to 20 ppm of *P. aeruginosa* strain PAR may trigger a type of protective mechanism, resulting in better growth than its control. This is certainly an interesting development that could have implications for further research in this area.

Additional research is required to identify microorganisms that have the ability to withstand the effects of fluoride. Gaining insight into the fundamental mechanisms that enable these microorganisms to tolerate fluoride could potentially provide us with a valuable resource for addressing fluoride contamination in various environments.

The observed decrease in germination rates under NaF stress is consistent with earlier research demonstrating fluoride’s inhibitory effects on seed germination. It has been observed that the germination percentages of tomato seeds decrease with increasing NaF concentrations, proving that they are sensitive to fluoride stress in a dose-dependent manner. The considerable improvement in germination rates in *P. aeruginosa* strain PAR-treated seedlings under NaF stress is notable. This shows that inoculating with *P. aeruginosa* strain PAR helps to lessen the negative impacts of NaF on seed germination, presumably through mechanisms like increased nutrition availability or stress tolerance induction. Chahine et al. (2023) reported that Jesca (African-Tanzania bean variety) seed germination considerably declined with higher levels of fluorides (3% NaF and 10% KF). Pelc et al. (2020) found that an upsurge in the fluoride concentration decreased seed germination, both in the roots and embryos of all tested cultivars of winter wheat.

The measurements of shoot height, leaf area, and root length provide a full assessment of the tomato plants’ overall growth and development. The decrease in these parameters under NaF stress demonstrates the deleterious influence of fluoride on plant morphology. However, under NaF stress, *P. aeruginosa* strain PAR-treated seedlings had higher shoot height, root length, and leaf area than uninoculated plants. This improvement could be related to the favorable effects of *P. aeruginosa* strain PAR on nutrient uptake, hormone production, or other growth-promoting mechanisms. Pant et al. (2008) evaluated the influences of 0.001, 0.01, and 0.02 M NaF on the seedling growth of Bengal gram (*Cicer arietinum* L.), wheat (*Triticum aestivum*), tomato (*Lycopersicon esculentum*), and mustard (*Brassica juncea*). As the treatment duration increased to 7 days, the growth of roots and shoots decreased along with the increasing concentration of fluorides. The analysis of root-shoot dry and fresh weight adds to the evidence that *P. aeruginosa* strain PAR has a favorable effect on plant biomass under NaF stress. The increase in dry and fresh weights in *P. aeruginosa* strain PAR-treated plants suggests that this PGPR can play a crucial role in easing the adverse impacts of NaF on plant productivity. This finding is critical to comprehend the practical implications of adopting *P. aeruginosa* strain PAR as a bioinoculant in fluoride-contaminated areas for sustainable agriculture. According to Chahine et al. (2023), high fluoride concentration reduced the growth and root-shoot biomass (95% and 50% with NaF and KF, respectively) of Jesca. Zulfiqar et al. (2023) reported that fluoride toxicity was reduced using fluoride-resistant bacterial strains (SS-10a and SS-5a) in the soil. Wheat plants were treated at varying sodium fluoride (NaF) concentrations, 0 (control), 150, 250, and 350 ppm, in a randomized full-block design. At 7 days after transplantation (DAT), fluoride-resistant bacteria were inoculated into the soil of two types of wheat, Ujala-15 and Faisalabad 2008. Fluoride stress had a significant negative impact on growth, biomass, and yield. However, the presence of fluoride-resistant bacteria helped the plants to cope with the stress and reduce the overall loss of productivity. The relative water content measurements provided information about the plants’ water status under NaF stress. Seedlings treated with *P. aeruginosa* strain PAR have exhibited an ability to maintain a higher relative water content compared to untreated plants. This suggests that the treatment has a positive impact on water uptake and retention, which could be beneficial for plant growth and development. This could be due to increased water efficiency or a decrease in transpiration rate, both of which contribute to the overall stress tolerance imparted by bacterial inoculation. *P. aeruginosa* strain has a notable capacity to build up soluble sugars, glycine betaine, and proline under NaF stress. The accumulation of glycine betaine within cells is known to provide protection to microorganisms under various abiotic stresses, either through osmoprotection or osmoregulation (Giri, 2011).

Our findings suggest a potential positive impact of *P. aeruginosa* strain PAR on mitigating the negative effects of NaF-induced stress on chlorophyll levels in tomato plants. According to Islam et al. (2014), bio inoculation of *P. aeruginosa* improved leaf chlorophyll content in wheat plants. According to Singh and Roychoudhury (2020), chlorophyll content decreased when seedlings were treated with two different NaF levels, 25 and 50 mg L^−1^.

The research indicates that while the presence of NaF in plants can lead to increased oxidative stress, there is a way to mitigate this negative impact. Inoculating with *P. aeruginosa* strain PAR can serve as a constructive measure to reduce the negative impact on MDA levels. Singh and Roychoudhury (2020) found that the seedlings faced oxidative damage when exposed to two different NaF amounts, i.e., 25 and 50 mg L^−1^, which resulted in increased F accumulation, inhibition in growth, and decreases in the content of lipid peroxidation (MDA content and activity of lipoxygenase). When plants are subjected to NaF stress, a by-product of this stress is the accumulation of high levels of reactive oxygen species (ROS). These ROS can be harmful to the plant and its growth. However, the plant has a natural defense mechanism in the form of antioxidative enzymes that work to detoxify the ROS and protect the plant from damage. Under 10 ppm, 20 ppm, 40 ppm, and 80 ppm NaF stress, we noticed a drastic fall in the activity of defensive antioxidant enzymes like GR, CAT, SOD, and APX in treated plants in comparison to non-inoculated plants.

Constantia and Ferniah (2020) found that several advantageous compounds like potassium, phosphates, zinc, silicon, and PGPR stimulate plant growth by fixing nitrogen, chelating iron, and other micronutrients. Plants inoculated with the *P. aeruginosa* strain PAR had increased Mg, K, P, and Fe content and decreased Na and F content compared to uninoculated plants. The present findings are consistent with the study of Islam et al. (2014), who revealed that *P. aeruginosa* treatment enhances the absorption of P and N in wheat plants. According to Sakthi Thesai et al. (2018), the *Bacillus flexus* (PN4) strain has the ability to produce drinking water that is free of fluoride and may also be used to create a new approach for bioremediation.

## 5 Conclusion

In conclusion, our findings highlight the significant potential of *P. aeruginosa* strain PAR as a bio-solution for alleviating fluoride-induced stress in tomato crop plants. Strain PAR’s robust features, as indicated by its considerable impact on a variety of physiological and biochemical parameters, highlight its efficacy as a PGPR. Strain PAR appears as a diverse ally in augmenting plant stress resilience, from promoting germination to altering antioxidant activities and nutrient levels. These findings not only add to our understanding of PGPR uses but also provide practical insights for sustainable agriculture in fluoride-contaminated soils. *P. aeruginosa* strain PAR stands out as a viable tool for farmers, providing a bio-solution to enhance crop resilience and productivity under fluoride-induced challenges.

## Data availability statement

The data presented in the study are deposited in the NCBI repository under accession numbers OR427951, OR427950 and OR427949.

## Author contributions

AS: Formal analysis, Investigation, Methodology, Validation, Writing – original draft, Writing – review & editing. AnP: Data curation, Investigation, Methodology, Writing – original draft, Writing – review & editing. MP: Investigation, Methodology, Writing – original draft, Writing – review & editing. SV: Project administration, Resources, Software, Validation, Writing – review & editing. RV: Project administration, Supervision, Validation, Visualization, Writing – review & editing. AA: Funding acquisition, Resources, Software, Validation, Writing – review & editing. HO: Formal analysis, Resources, Software, Validation, Writing – review & editing. LR: Data curation, Formal analysis, Methodology, Validation, Writing – review & editing. NE: Data curation, Formal analysis, Resources, Software, Writing – review & editing. VY: Visualization, Writing – review & editing, Investigation, Methodology, Supervision. DS: Data curation, Funding acquisition, Project administration, Supervision, Writing – review & editing. RC: Data curation, Project administration, Resources, Visualization, Writing – review & editing. AsP: Conceptualization, Supervision, Visualization, Writing – original draft, Writing – review & editing.
